# Mesenchymal Stem Cells Loaded with Gelatin Microcryogels Attenuate Renal Fibrosis

**DOI:** 10.1155/2019/6749326

**Published:** 2019-10-31

**Authors:** Xiaodong Geng, Quan Hong, Kun Chi, Shuqiang Wang, Guangyan Cai, Di Wu

**Affiliations:** ^1^Department of Nephrology, Chinese PLA General Hospital, Chinese PLA Institute of Nephrology, State Key Laboratory of Kidney Diseases, National Clinical Research Center for Kidney Diseases, Beijing Key Laboratory of Kidney Diseases, 28 Fuxing Road, Beijing 100853, China; ^2^Chinese Traditional and Western Medicines Combination Center for Kidney Diseases Treatment, Beidaihe Rehabilitation and Recuperation Center, Chinese People's Liberation Army Joint Logistics Support Force Qinhuangdao, Qinhuangdao 066100, China

## Abstract

**Background:**

The treatment of chronic kidney diseases (CKDs) by different approaches using mesenchymal stem cells (MSCs) has made great strides. In this study, we aimed to explore the potential mechanism of gelatin microcryogels (GMs) as a cell therapeutic vector to block the progression of CKD.

**Methods:**

In vivo, the pedicled omentum valve with MSC-loaded GMs was packed onto 5/6 nephrectomized kidneys derived from rats. The therapeutic effects were evaluated. In vitro, TNF-*α*, TGF-*β*, and MSCs were added to the medium of the HK-2 cell culture system, and key genes involved in anti-inflammatory and antifibrosis effects were evaluated by qPCR.

**Results:**

After 12 weeks of MSC transplantation, kidney functions, such as serum creatinine, urea nitrogen, and 24-hour urine protein, were significantly improved. The pedicled omentum valve was packed with MSC-loaded GMs onto the 5/6 nephrectomized kidney, and the expressions of collagen IV, *α*-SMA, and TGF-*β* were all evaluated by immunohistochemical staining and western blot analysis. MSC-loaded-GMs also showed antifibrotic effects by inducing the upregulation of HO-1, BMP-7, and HGF and the downregulation of MCP-1 at the mRNA level. Four weeks after MSC-loaded GM treatment, we found that the mRNA levels of TNF-*α* and IL-6 were clearly reduced. MSC-conditional medium (MSC-CM) showed that the TNF-*α*-induced expression of IL-8 and IL-6 mRNA was reversed; E-cadherin mRNA was upregulated; and the TGF-*β*-induced expression of collagen IV, *α*-SMA, and fibronectin (FN) mRNA in HK-2 cells was reduced.

**Conclusions:**

We demonstrated that the pedicled omentum valve packed with MSC-loaded GMs had a renal protective effect on the 5/6 nephrectomized kidney by observing the anti-inflammatory and antifibrosis effects.

## 1. Introduction

With the increasing incidence of obesity, diabetes, and cardiovascular diseases, the prevalence rate of chronic kidney disease (CKD) is gradually increasing. Most end-stage renal disease patients can survive only on long-term dialysis or kidney transplantation. Recent advances in stem cell biology have introduced the possibility for regenerating kidneys [1]. Mesenchymal stem cells (MSCs) have a lower immunogenicity than other stem cells, so they are widely used in various research and clinical practices of allogeneic cell therapy. According to the results of Tögel et al. [2], MSCs can inhibit the release of many proinflammatory factors and activate anti-inflammatory factors, thus playing a protective role in the renal function of rats with acute renal injury. No differentiation of MSCs into tubular epithelial cells was found in this process. MSCs may also exhibit immunoregulatory properties. A large number of experiments in vivo or in vitro have confirmed that MSCs can significantly inhibit the proliferation and activity of monocytes, macrophages, lymphocytes, and other immune cells. This property provides immunosuppressive and anti-inflammatory activity for immune-mediated diseases [3].

Multiple studies have shown that the therapeutic effect of MSCs on acute renal failure is exciting [4]. Immunomodulatory and antiapoptotic effects are related to the pathophysiological process of renal disease improvement, suggesting that MSCs may play a role through the paracrine mechanism [5]. In our previous studies, we used gelatin microcryogels (GMs) as cell carriers for stem cells. GMs are biocompatible and biodegradable materials. We showed that the pedicled greater omentum flap packed with MSC-loaded GMs on 5/6 nephrectomized kidneys could effectively block the development of CKD, indicating that this method may become a new direction for the treatment of CKD in the future [6]. MSCs are associated with the ability to repair kidney damage, and the mechanism of their remarkable renovation of renal function can be explained by cell secretion and other factors. In this study, we used MSC-loaded GM treatments to test the effects of MSCs on the progression of CKD and tried to clarify the mechanism of MSCs in the progression of antifibrosis in CKD.

The infiltration of inflammatory cells into the interstitial and glomerular regions is a hallmark of CKD. Here, we used MSC-loaded GM treatments to interrupt renal tissue fibrosis in CKD. We examined whether MSC-loaded GMs could also aid in the construction of an animal model of CKD and elaborate on the potential mechanism of this strategy. We demonstrated that the positive effects of MSCs on CKD mainly depend on antifibrosis and anti-inflammatory effects.

## 2. Materials and Methods

### 2.1. MSC Culture

SD (Sprague-Dawley) rat bone marrow-derived MSCs were purchased from Cyagen Biosciences (Cyagen Biosciences, Sunnyvale, CA, USA). All manufacturer instructions were strictly followed.

The supplier tested the cell surface phenotypes used in the experiment. They were CD44+, CD29+, CD90+, CD34−, CD45−, and CD11b−. The certificate of analysis for MSCs can be found in the supplemental material. Bone marrow mesenchymal stem cells (BMSCs) grow in a 25 cm^2^ culture flask. The culture flask is placed in a constant temperature and humidity incubator. The culture conditions were set at 37°C, 5% CO_2_, and 90% humidity. The medium used for culturing cells in vitro is replaced every 12 hours. The cells grow and divide steadily in the sixth to eighth passages of MSCs, which can be used in subsequent experiments.

### 2.2. Experimental Animals and Procedures

Male SD rats (220–250 g) were provided by the Experimental Animal Center of the Academy of Military Medical Sciences. Rats were fed standard rodent feed and drinking water. The rats were preadapted for 7 days before the start of the study and then placed in a constant-temperature feeding room. An artificial light source with a 12-hour light/dark cycle was set up. Briefly, the rats were operated on under general anesthesia to remove the upper and lower poles of the right kidney and left kidney so that 5/6 of the kidney tissues was removed. The established CKD model of rats did not induce renal hypertension, and all the conditions met the experimental requirements. We designed the pedicled greater omentum flap packed with GMs or cells on remnant renal tissue to induce healing with the remaining kidney tissue.

The following groups were evaluated: Sham, only removal of renal capsule; NPX, 5/6 resection of kidney; NPX + MSC, pedicled omentum valve packed with MSC-loaded GMs on the 5/6 nephrectomized kidney. Each experimental group comprised 8 rats. The rats in each experimental group were euthanized at 12 weeks after the establishment of animal models. After euthanasia, the remaining kidney tissue and the fused omentum, or the integrity of the surrounding omentum, needed to be maintained before biochemical treatment of the kidney and its surrounding tissues to carry out subsequent histological experiments.

### 2.3. Cell Autoloading into GMs and Cell Viability Assessment

GMs were provided by the cell research center of the Biomedical Engineering Department of Tsinghua University. After cleaning and drying, the loaded cells were sterilized using the ethylene oxide sterilization system, i.e., GMs fully activated with ethylene oxide gas for 12 hours, followed by vacuum exhaust for 12 hours (AN74j/Anprolene; Anderson Sterilization). According to previous research results, MSCs were cultured and isolated in a special growth medium. Subsequently, the 60 *μ*l MSC suspension (initial cell inoculation density of 8 × 10^6^ cells/ml) was transfused to 600 GMs, which were tightly packed, and the stem cells were automatically absorbed into porous hydrates. Subsequently, GMs were placed in the humidifying chamber and incubated at 37°C for 2 hours to make the stem cells fully adhere. Subsequently, MSC-loaded GMs were cultured in the medium for 2 days. MSC-loaded GMs were then fixed on 5/6 nephrectomy sections using pedicled greater omental flaps.

### 2.4. Measurement of Renal Function and Chronic Kidney Disease Evaluation

The weight of the rats in each experimental group was measured weekly. At 0, 4, 8, and 12 weeks after the experiment, 24-hour urine and blood samples were collected for detection. During 24-hour urine collection, rats were placed in metabolic cages that lacked food but were able to obtain adequate drinking water. Urine was collected on antibiotic/antifungal solution (Sigma, St. Louis, MO; A5955) and stored in a refrigerator at −80°C. Blood samples were obtained through the caudal vein. Serum urea and creatinine (CR) levels in rats were measured by using a Hitachi 7150 automatic biochemical analyzer. The urinary protein was determined by the Coomassie blue method.

### 2.5. Histopathological Examination

The kidney tissue was fully immersed in 4% formalin solution for 24 hours and then dehydrated and embedded. The kidney section thickness was 3 *μ*m and then stained with periodic acid-Schiff (PAS) and Masson's trichrome. The degree of renal histological injury (glomerulosclerosis and tubular injury) was assessed with a semiquantitative scale developed by Cao et al. [7]. The evaluation process was carried out by the blind method. The degree of glomerular injury was evaluated by observing renal tissue sections with a 200-fold magnification optical microscope: Class 0, no abnormal manifestations; Class 1, less than 25% of the glomerulus was involved, mild glomerular injury and mesangial dilatation; Class 2, mild sclerosis occurred in 25%–50% of the glomerulus; Class 3, moderate glomerulosclerosis occurs in 50%–75% of the glomerulus; Class 4, more than 75% of the glomeruli developed severe sclerosis. The degree of renal tubular injury was observed under a 100-fold microscope. Level 0, no abnormal manifestations; Class l, renal interstitial inflammation, fibrosis, local tubular dilatation, and casting were observed in less than 25% of the visual field; Class 2, lesions occurred in 25%–50% of the areas in the visual field; Class 3, lesions occurred in 50%–75% of the areas in the visual field; and Class 4+, lesions occurred in more than 75% of the areas in the visual field. The average score of rats in each experimental group was used as the glomerulosclerosis index and renal tubular injury rating.

### 2.6. Real-Time PCR

TRIzol (Abcam, USA) was used to isolate the total intracellular RNA. The cDNA was generated using a reverse transcription kit (Applied Biosystems, Foster City, CA, USA) in accordance with the instructions. The relative expression of the target gene was calculated by using the 2^−ΔΔCT^ method. The sequence of primers used in the experiment is shown in Table 1.

### 2.7. Western Blot Analysis

Mouse anti-*β*-actin monoclonal antibody was obtained from Sigma (St. Louis, MO, USA). Rabbit polyclonal anti-TGF-*β*, rabbit polyclonal anti-*α*-smooth muscle actin (SMA), horseradish peroxidase-labeled goat anti-rabbit immunoglobulin G (IgG), rabbit polyclonal anti-type IV collagen, rabbit polyclonal anti-TGF-*β*, rabbit anti-Smad2/3 antibody, rabbit anti-pSmad2/3 antibody and horseradish peroxidase-labeled goat anti-rabbit IgG were purchased from Abcam (Cambridge, MA, USA). The spare kidney tissue was removed and thawed and homogenized for radio immunoprecipitation. The homogenate was centrifuged for 10 minutes at 12 000 rpm in a 4°C cryogenic high-speed centrifuge, and then, the supernatant was collected. Quantitative 70 *μ*g protein samples were prepared by gel electrophoresis and then transferred to a cellulose acetate membrane. A 1x casein solution was used to block the membranes for 1 hour after transfer. After blocking, the membrane was transferred to the refrigerator and stored at 4°C and incubated overnight with the first antibody. Then, the samples were washed thoroughly and incubated with the second antibody. Images of protein imprinting were analyzed using Image J 1.42 software after antibody incubation.

### 2.8. Immunohistochemistry

Kidney tissues were prepared into slices. After antigen detection, rabbit polyclonal anti-TGF-beta, rabbit anti-alpha-SMA, and rabbit polyclonal anti-type IV collagen were used for labeling at 4°C overnight. Then, they were incubated with a secondary antibody (Dako, Glostrup, Denmark), and the sections were treated with an avidin-biotin peroxidase conjugate. Finally, the sections were stained with heme using DAB color reagent (Dako) and observed under a microscope.

### 2.9. HK-2 Cell Cultures, Preparation of MSC-CM, and Coculture with MSC-CM

Human proximal tubular cells (HK-2 cells) were purchased from the American Type Culture Collection (Manassas, VA) and cultured in Dulbecco's Modified Eagle Medium (containing 10% FBS and antibiotics). Cell culture flasks were placed in a constant temperature and humidity incubator in an atmosphere of 5% CO_2_ and 95% air at 37°C. MSCs were cultured in vitro as previously described. The supernatant of the culture system was collected in the form of MSC-CM, filtered by a 0.22-micron membrane filter (Millex-GS 33 mm, Millipore), and sealed and stored in a refrigerator at −80°C until further use. To investigate whether conditioned MSCs would attenuate inflammation induced by TNF-*α* and inhibit fibrosis induced by TGF-*β* in HK-2 cells, conditioned MSCs were cocultured with HK-2 cells treated with recombinant human TNF-*α* (10 ng/ml) or TGF-*β* (15 ng/ml).

### 2.10. Quantification of Serum Cytokine Level

The expression of human MCP-1 (R&D system) protein in vitro was determined by ELISA. The procedure was strictly carried out according to the instructions.

### 2.11. Statistical Analysis

Excel 2017 was used to organize the data of each experimental group, and the data were expressed as the mean ± standard deviation. Data difference analysis between groups was performed using SPSS 21.0 statistical software using Student's *t*-test. A *P* < 0.05 was used to indicate significant differences between the groups.

## 3. Results

### 3.1. Pedicled Greater Omentum Packed with MSC-Loaded GMs on the Injured Kidney Ameliorates Renal Dysfunction

**Figure 1:**
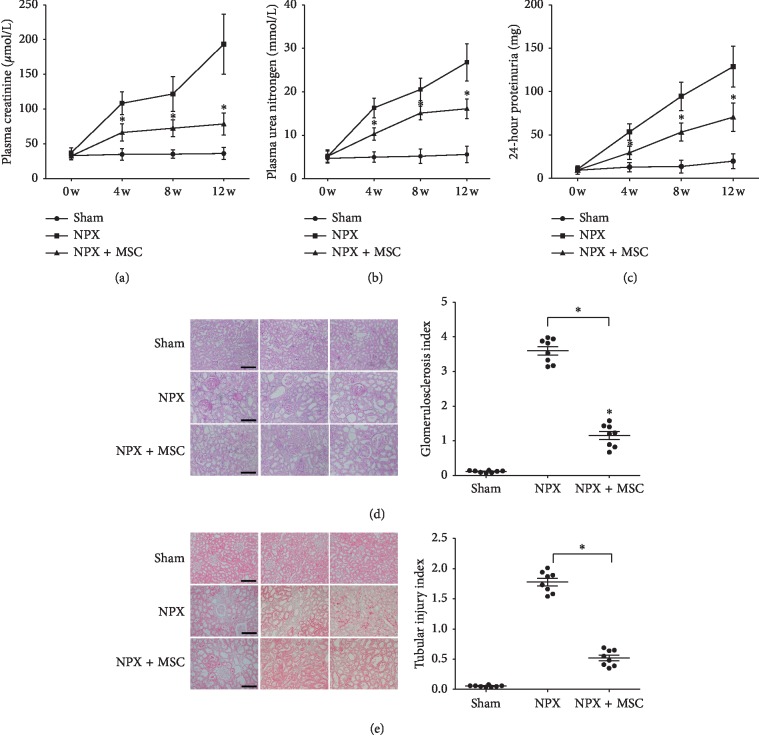
Effects of MSC-loaded GMs on rat renal function indexes, renal histology, and tubular injury scores. Serum creatinine (a), BUN values (b), and 24-hour proteinuria (c) at 0, 4, 8, and 12 weeks after MSC transplantation. Kidney histology showing different grades of glomerular damage ((d) in PAS staining) and tubular injury ((e) in Masson's trichrome staining) observed at week 12 after inducing chronic kidney disease in rats. Sham, Sham group; NPX, 5/6 nephrectomy group; NPX + MSC, 5/6 nephrectomy and pedicled greater omentum packed with MSC-loaded GMs on remnant renal tissue group. ^*∗*^*P* < 0.05 versus NPX group (*n* = 8) (scar bar, 200 *μ*m; magnification, 200X).

We analyzed the renal functional parameters of different groups. The NPX + MSC group with the pedicled great omentum packed with MSC-loaded GMs on the injured kidney protected renal function by lowering serum blood urea nitrogen (BUN) and CR levels. Compared with the control group, the levels of BUN and CR in the NPX group increased significantly at 0, 4, 8, and 12 weeks (*P* < 0.05). Compared with the NPX group, the levels of CR and BUN in the NPX + MSC group decreased significantly at 4, 8, and 12 weeks after BMSC transplantation (*P* < 0.05) (Figures 1(a) and 1(b)). During the study, we compared and analyzed the changes in 24-hour proteinuria in rats. The results showed that 24-hour proteinuria in the NPX group increased compared with the control group (*P* < 0.05). The 24-hour proteinuria in the NPX + MSC group was significantly lower than that in the NPX group (*P* < 0.05) (Figure 1(c)).

The quantification of fibrosis and glomerulosclerosis are hallmarks of the 5/6 NPX rat model. The dynamic changes of glomerular and tubular injury were very obvious in the NPX + MSC group. In the NPX + MSC group compared with the NPX group, the degree of glomerulosclerosis was reduced (PAS staining in Figure 1(d)). Masson's trichrome staining showed that the fibrosis area of the MSC-treated group was significantly reduced (Figure 1(e)).

### 3.2. MSC-Loaded GMs Showed an Antifibrotic Effect

**Figure 2:**
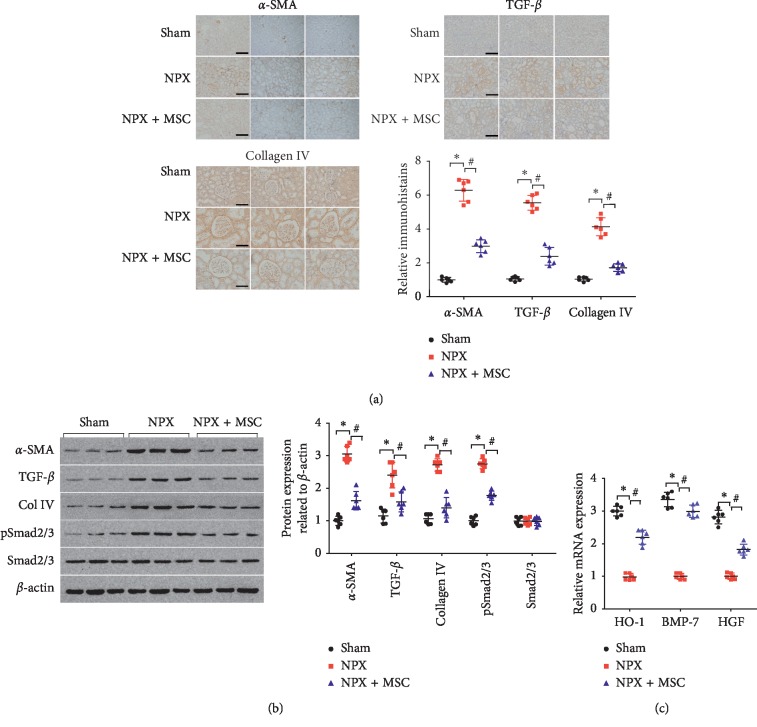
Antifibrotic effect of MSC-loaded GMs at 12 weeks. (a) Expression of *α*-SMA, TGF-*β*, and collagen IV in kidney tissues of the Sham, NPX, and NPX + MSC groups stained by immunohistochemistry. The magnification for *α*-SMA and TGF-*β* is 200X (scale bar, 200 *μ*m); the magnification for collagen IV is 400X (scale bar, 100 *μ*m). (b) Representative western blot and relative bar graph analysis of *α*-SMA, TGF-*β*, and collagen IV protein levels in the Sham, NPX, and NPX + MSC groups. (c) Antifibrotic molecule of HO-1, BMP-7, and HGF mRNA expression in kidney tissue. Sham, sham group; NPX, 5/6 nephrectomy group; NPX + MSC, 5/6 nephrectomy and pedicled greater omentum packed with MSC-loaded GMs on remnant renal tissue group. ^*∗*^*P* < 0.05 versus Sham group; ^#^*P* < 0.05 versus NPX group (*n* = 6).

Staining for *α*-SMA, TGF-*β*, and collagen IV was proportional to the severity of chronic renal failure (CRF). To examine the expression of *α*-SMA, TGF-*β*, and collagen IV in the renal tissues, immunohistochemical staining was performed. Immunohistochemical staining indicated that low levels of *α*-SMA, TGF-*β*, and collagen IV were detected in the renal tissues of the rats in the Sham group. However, there was increased deposition of *α*-SMA, TGF-*β*, and collagen IV at 12 weeks in the NPX group (Figure 2(a)). The immunohistochemical staining of *α*-SMA, TGF-*β*, and collagen IV was reduced in renal tissues from the rats treated with MSC-loaded GMs in the NPX + MSC group compared with the NPX group (*P* < 0.05). Since TGF-*β*/Smad signaling plays a critical role in renal fibrosis and TGF-*β* exerts its biological effects by activating Smad 2/3, we examined the protein levels of *α*-SMA, TGF-*β*, collagen IV, Smad 2/3, and pSmad 2/3. Western blot analysis demonstrated that the protein levels of *α*-SMA, TGF-*β*, collagen IV, and pSmad2/3 were significantly increased in the NPX group compared with the Sham group. The expression levels of *α*-SMA, TGF-*β*, collagen IV, and pSmad2/3 were significantly decreased in the NPX + MSC group compared with the Sham group, which was reversed by treatment with MSC-loaded GMs (Figure 2(b)).

Under the action of heme oxygenase-1 (HO-1), bone morphogenetic protein-7 (BMP-7), and hepatocyte growth factor (HGF), renal fibrosis can be blocked or even reversed. Therefore, in the course of the study, we observed the above indicators. The results showed that the expression levels of HO-1, BMP-7, and HGF in 5/6 renal tissues were significantly decreased in the NPX group. Compared with the NPX group, the expression levels of HO-1, BMP-7, and HGF in the NPX + MSC group increased significantly again (*P* < 0.05) (Figure 2(c)).

### 3.3. Effects of MSC-Loaded GMs on Renal Cortical Anti-Inflammatory Cytokine Expression In Vivo

MSC-loaded GMs showed antifibrotic effects at 12 weeks after 5/6 nephrectomy. The role of inflammation in the progression of CKD is well known. We suspected that the anti-inflammatory mechanism could play an important role in the early stage of CRF. To verify this, rats were subjected to the remnant model and treated with MSC-loaded GMs. We sacrificed some rats to study the mechanism in this process at 4 weeks.

**Figure 3:**
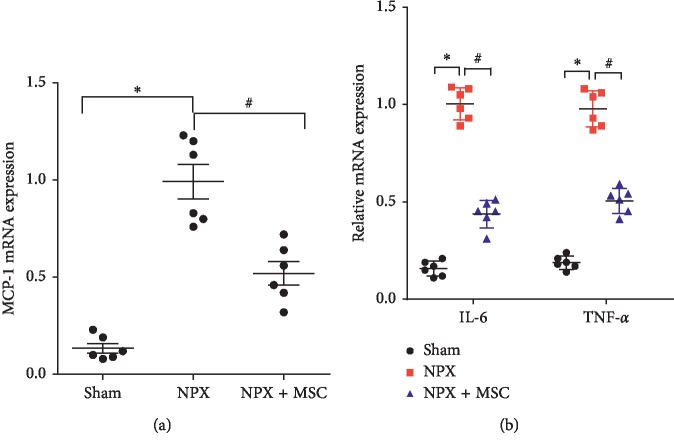
Inflammatory molecule mRNA expression in rat kidney tissue at 4 weeks. Gene expression in kidneys of NPX and NPX + MSC groups. (a) MCP-1 mRNA expression. (b) IL-6 and TNF-*α* mRNA expression. The kidney tissue was treated just once and followed for 4 weeks. Data are expressed as the mean of 2^−ΔΔCT^; ^*∗*^*P* < 0.05 versus Sham group; ^#^*P* < 0.05 versus NPX group (*n* = 6).

Under the action of various cytokines and chemokines of inflammatory cells, many inflammatory cells, such as lymphocytes, macrophages, and monocytes, aggregate in the remnant kidney tissue. Inflammatory influx in remnant kidney tissue is a key factor for the continuous decline in renal function. Monocyte chemoattractant protein-1 (MCP-1) has a very important effect on the aggregation of monocytes and macrophages during inflammation. The results showed that the expression of MCP-1 was significantly inhibited in remnant kidney tissues after treatment with MSC-loaded GMs (Figure 3(a)). At the same time, real-time quantitative PCR was used to check the expression of TNF-*α* and IL-6. The results showed that compared with the NPX group, the gene expression levels of TNF-*α* and IL-6 in MSC-loaded GMs significantly decreased (Figure 3(b)).

### 3.4. Anti-inflammatory and Antifibrotic Effects of MSC-CM on Proximal Tubular Cells In Vitro

**Figure 4:**
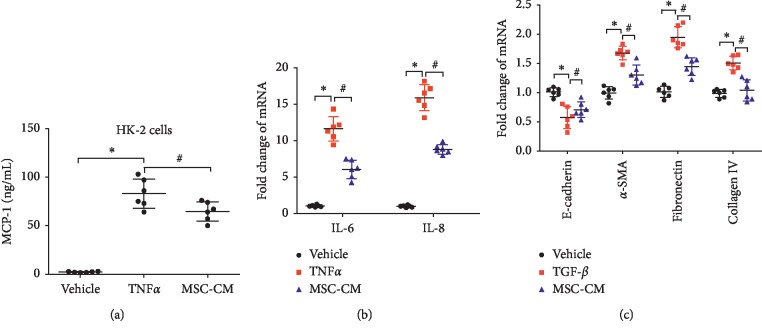
Anti-inflammatory and antifibrotic effects of MSC-CM on proximal tubular cells in vitro. (a) Effects of MSC-CM on TNF-*α*-induced MCP-1 production in HK-2 cells. After 24 hours of culture, MCP-1 levels in the supernatant of HK-2 cells were measured using ELISA. ^*∗*^*P* < 0.05 versus vehicle medium; ^#^*P* < 0.05 versus HK-2 cells treated with TNF-*α* (*n* = 6). (b) Proinflammatory cytokine in HK-2 cells was assessed by real-time PCR after a 24-hour incubation. ^*∗*^*P* < 0.05 versus vehicle medium; ^#^*P* < 0.05 versus HK-2 cells treated with TNF-*α* (*n* = 6). (c) HK-2 cells were cocultured with MSC-CM in the presence of TGF-*β* for 3 days for E-cadherin, *α*-SMA, fibronectin, and collagen IV mRNA detection by real-time PCR. ^*∗*^*P* < 0.05 versus HK-2 cells exposed to vehicle medium; ^#^*P* < 0.05 versus HK-2 cells treated with TGF-*β* (*n* = 6).

To further clarify the anti-inflammatory mechanism of MSC in vitro, we next investigated the effects of MSC-conditional medium (MSC-CM) on TNF-*α*-induced MCP-1 production in proximal tubular cells (HK-2 cells). During the culture of HK-2 cells in vitro, more MCP-1 was produced by TNF-*α* stimulation than by single-medium treatment. HK-2 cells were stimulated with TNF-*α* (10 ng/ml) and then cocultured with MSC-CM for 24 hours. The MCP-1 protein content in the supernatant of the culture system was determined. Stimulation with TNF-*α* alone increased MCP-1 production in HK-2 cells compared with cells treated with medium alone. Treatment with MSC-CM dramatically decreased TNF-*α*-mediated MCP-1 production compared with vehicle pretreatment in HK-2 cells (Figure 4(a)). These results suggest that MSC-CM can affect the secretion of MCP-1, which may be related to the decrease in serum MCP-1 levels in MSC-loaded GM rats.

MSC-CM can reduce the production of MCP-1 mediated by TNF-*α*. We measured the changes in IL-6 and IL-8 expression in HK-2 cells induced by TNF-*α* after MSC-CM treatment. After pretreatment with MSC-CM, HK-2 cells were exposed to TNF-*α* (10 ng/ml), and the expression levels of IL-6 and IL-8 were detected. Although TNF-*α*-stimulated HK-2 cells significantly increased the expression of IL-8 and IL-6, the expression of IL-8 and IL-6 decreased significantly after MSC-CM treatment (*P* < 0.05) (Figure 4(b)).

MSC-CM exhibited an anti-inflammatory mechanism in HK-2 cells that underwent a short-term TNF-*α* treatment. Long-term incubation with TGF-*β* could induce tubular epithelial mesenchymal transition (EMT). We suspected that MSC-CM could also induce an antifibrotic effect in TGF-*β*- (15 ng/ml) treated HK-2 cells. The effect of MSC-CM on TGF-*β*-induced tubular EMT was examined. HK-2 cells treated with TGF-*β* were cocultured with MSC-CM for 3 days. The results showed that the EMT process of renal tubular epithelial cells was blocked or reversed, and the expression of E-cadherin was upregulated, while the upregulation of *α*-SMA, FN, and collagen IV induced by TGF-*β* decreased again (Figure 4(c)).

## 4. Discussion

At present, the development of cell tissue engineering and regenerative medicine is particularly rapid. Many scholars are committed to applying this theory to the treatment of renal failure [8]. Because cell therapy is relatively simple to perform, stem cells isolated from their own tissues have fewer side effects in the course of treatment, and its clinical application value is particularly extensive [9]. Our previous study showed an effective method of stem cell treatment for CKD. We used the pedicled greater omentum flap to wrap the stem cell-loaded GMs onto the 5/6 nephrectomized rat kidney to prevent stem cell leakage and to allow the stem cells to survive in the body, creating an optimal in vivo targeted therapy pathway and improving the clinical effect of cell therapy [6]. GMs can form biomimetic 3D ecological structures in local tissues, enrich extracellular matrix (ECM), and make the basic processes of cell growth, proliferation, and differentiation proceed more smoothly [10]. The main component of the omentum is loose connective tissue, which is rich in blood and lymphatic circulation. The pedicled greater omentum flap is more conducive to promoting local vascular regeneration of injured tissues and organs, improving immunity, and promoting healing and functional recovery of injured tissues [11].

The strong anti-inflammatory, antiapoptotic, and antifibrotic effects of MSCs have been confirmed. In the treatment of renal failure, the improvement of renal function is closely related to the paracrine effect of MSCs, which inhibits local inflammatory response and fibrosis [12]. Therefore, we selected MSCs for stem cell therapy at this time.

Transfection of MSCs loaded into GMs is simple. The results showed that the function of the residual kidney was significantly improved after pedicled greater omentum flaps loaded with MSC-loaded GMs were applied to the 5/6 nephrectomized kidneys. In the control group, 4 weeks after 5/6 nephrectomy, CKD appeared in rats, which was characterized by increased urinary protein and serum CR/urea nitrogen levels. However, in the NPX + MSC group with pedicled greater omentum flaps with MSC-loaded GMs, the plasma CR/urea nitrogen level did not increase significantly and glomerulosclerosis and tubulointerstitial injury were also mild.

Kidney fibrosis is characterized by fibroblast proliferation, myofibroblast differentiation, and deposition of ECM in the renal tissue. TGF-*β* is a key fibrogenic cytokine that plays a critical role in the expression of *α*-SMA and the induction of kidney fibrosis [13]. *α*-SMA is the hallmark of myofibroblasts, which are generally considered to be the key effector cells in the development of fibrosis [14]. Our results showed that MSC-loaded GM treatment could attenuate kidney fibrosis progression through downregulating the expression of TGF-*β* and *α*-SMA. Changes in the concentration of ECM components may reflect the degree of kidney fibrosis. Type IV collagen is a major component of basement membranes [15]. We analyzed the expression of a fibrous ECM component, type IV collagen, by immunohistochemical analysis. The results showed that the expression of collagen IV in the MSC-loaded GM treatment group was decreased compared with the NPX group. TGF-*β* has been considered as a main mediator in renal fibrosis and induces renal scarring largely by activating its downstream Smads signaling pathway via canonical or noncanonical pathway. Our results showed that phosphorylated Smad 2/3 is significantly lower in the MSC treatment group than the NPX group. This indicated that MSCs prevented TGF-*β* expression then decreased Smad 2/3 from phosphorylation, finally attenuated the Smad 2/3-dependent renal fibrosis in chronic kidney diseases.

MSCs also have antifibrotic properties. Fibrosis is an important phenomenon in the process of CRF. The expression changes of antifibrosis molecules are very significant in nephrectomized rats. Compared with the nephrectomy group, the expression of HO-1, BMP-7, and HGF increased in MSC-loaded GM-treated rats. HGF can bind to MET, which is a transmembrane receptor of the tyrosine kinase superfamily, and its inhibitory effects on tissue fibrosis have been confirmed in various research models, such as residual kidney, unilateral ureteral obstruction, and diabetic nephropathy. HO-1 can transform heme into carbon monoxide, iron, and biliverdin (eventually bilirubin) enzymes in vivo. Current studies have shown that HO-1 has a protective and reversal function against multiple tissue injuries and organ dysfunction. A large number of studies have confirmed that HO-1 deficiency has a significant relationship with the spread of inflammation in renal tissue, the occurrence of fibrosis in renal lesions, and the expression of TGF-*β* in renal tubular cells [16]. Therefore, the high expression of HO-1 in the kidney tissues of rats treated with MSC-loaded GMs suggests that the progression of renal fibrosis will be inhibited. BMP-7 is a member of the TGF-*β* superfamily and a 35 kDa homologous secretory protein. The protective effect of BMP-7 on renal function in various kidney diseases is closely related to pathophysiological processes, such as antagonizing TGF-*β*-dependent fibrosis, reducing apoptosis of renal tubular epithelial cells and podocytes, and alleviating inflammation caused by the anti-TGF-*β* mechanism [17].

The evolution and outcome of inflammation play an important role in all chronic diseases. The results showed that treatment with MSC-loaded GMs could effectively inhibit the gene expression and protein secretion of IL-6 and TNF-*α* in renal tissue, thus achieving an anti-inflammatory effect. MSCs have powerful functions in vivo, and the immune regulation ability is very obvious. The extensive glomerulosclerosis in the remnant kidney model may be closely related to immune dysregulation and abnormal activation of the renin-angiotensin system [18]. In addition, the growth factor BMP-7 plays an irreplaceable role in the repair and regeneration of kidney tissue [19]. The results showed that BMP-7 expression in renal tissue was significantly increased 12 weeks after CRF was treated with MSC-loaded GMs. Thus, the early attenuation of inflammation could imply a reduction in later fibrosis activity.

To further clarify the effects of MSC anti-inflammation and antifibrosis, we cocultured TNF-*α*-treated HK-2 cells with MSC-CM for 24 hours and TGF-*β*-treated HK-2 cells with MSC-CM for 3 days in vitro. The results showed that TNF-*α* induced HK-2 cells to produce more IL-6 and IL-8, and then, when they were cocultured with MSCs, the levels of IL-6 and IL-8 decreased significantly. MSC-CM can significantly inhibit the overexpression of MCP-1 in HK-2 cells mediated by TNF-*α*. These data fully indicate that MSC-CM can improve renal function and reverse renal tissue damage through its powerful anti-inflammatory effect. The results of this study suggest that MSC-CM can inhibit or reverse the fibrosis of damaged kidney tissue, and its specific mechanism may be related to the inhibition of the expression of *α*-SMA, FN, and collagen IV. In conclusion, MSCs have shown unique therapeutic effects in the treatment of CRF due to their strong anti-inflammatory and antifibrotic effects and have broad prospects for clinical application.

For future study design, we have to notice that our current study has some limitations. However, we showed that MSC-loaded GMs can attenuate fibrosis progression in the rat kidney remnant model. Other animal models need to be designed to examine the effects of MSC-loaded GMs methods. In our experiment, we injected the MSC-loaded GMs during the surgery, and this may not be possible to distinguish between preventive approach and reversal treatment. Future research should be designed for distinguishing the differences. In addition, we used only a single dose administration of MSC-loaded GMs in the surgery and did not observe the effect of repeated administration on the antifibrosis. Thus, future studies should evaluate dose responses and explore different timings of administration.

## 5. Conclusions

Taken together, our data clearly demonstrated that MSC-loaded GMs can enhance the expression of antifibrotic molecules and attenuate fibrosis progression in the remnant model kidney. We provide new data to support the significant antitubular inflammation and renal fibrosis effects of MSCs in the treatment of CKD. This is likely mediated by the paracrine action of MSCs. In summary, the pedicled great omentum flaps packed with MSC-loaded GMs on the 5/6 nephrectomized kidneys could slow the progression of CKD. We also speculate that early-stage modulation of the immune response could reduce later fibrosis. The potential anti-inflammatory and antifibrosis effects of MSCs warrant confirmation in other animal models of CKD.

## Figures and Tables

**Table 1:** Primer sequences used in real-time PCR.

Genes	Forward (5′ ⟶ 3′)	Reverse (5′ ⟶ 3′)
HO-1 (rat)	GTAGAGGCGGCTGTTCTGAG	ATCAAAGTGGCCATGACGCT
BMP-7 (rat)	TAATTCGGCGCCCATGTTCA	GGCCTTGTAGGGGTAGGAGA
HGF (rat)	ACAGCTTTTTGCCTTCGAGC	GCTTGTGAAACACCAGGGTC
MCP-1 (rat)	TGATCCCAATGAGTCGGCTG	TGGACCCATTCCTTATTGGGG
IL-6 (rat)	AGCGATGATGCACTGTCAGA	GGAACTCCAGAAGACCAGAGC
IL-6 (human)	TCAATATTAGAGTCTCAACCCCCA	TTCTCTTTCGTTCCCGGTGG
IL-8 (human)	TGGACCCCAAGGAAAACTGG	GTTTGCTGTGCTTCTCTTGGAT
TNF-*α* (human)	GCCCTACTATTCAGTGGCGA	GCTTCTTCCCACCCACAAGA
E-cadherin (human)	ATGCTGATGCCCCCAATACC	ATCTTGCCAGGTCCTTTGCT
*α*-SMA (human)	CTTGTTTGGGAAGCAAGTGGG	GATTCCTGACAGTGCTTGGC
FN (human)	ACAAGCATGTCTCTCTGCCA	CCAGGGTGATGCTTGGAGAA
Collagen IV (human)	GACCGAGCCCTACAAAACCC	CTCAGGCTCTCAGGCCAC
GAPDH	GTAACCCGTTGAACCCCATT	CCTACCAACGGTAGTAGCG

PCR: polymerase chain reaction.

## Data Availability

The data sources used to support the findings of this study are included within the article.
